# Metabolomic signatures prior to in-hospital cardiac arrest: insights into potential therapeutic targets

**DOI:** 10.1038/s41598-026-56981-w

**Published:** 2026-06-12

**Authors:** Taline Lazzarin, Julia S. Nunes, Raquel S. Ballarin, Paula S. Azevedo, Filipe W. L. Pereira, Sergio A. R. de Paiva, Suzana E. Tanni, Leonardo Zornoff, Rafael Garrett, Daryl Jones, Marina A. Alves, Marcos F. Minicucci

**Affiliations:** 1 Medical School, Internal Medicine Department, São Paulo State University (Unesp), Botucatu, Brazil; 2 Federal University of Rio de Janeiro, Rio de Janeiro, Brazil; 3 Australian and New Zealand Intensive Care Research Centre (ANZIC-RC), School of Public Health and Preventive Medicine, Monash University, Melbourne, Australia; 4 Internal Medicine Department - Botucatu Medical School, UNESP, Rubião Junior s/n, Botucatu, CEP: 18618-970 SP Brazil

**Keywords:** Cardiac arrest, Metabolomics, Biomarkers, Critical care, Resuscitation, Biomarkers, Cardiology, Diseases, Medical research

## Abstract

Plasma metabolomic alterations occurring up to 48 h before in-hospital cardiac arrest (IHCA) and their associations with return of spontaneous circulation (ROSC) and in-hospital mortality (IHM) were evaluated in this exploratory case-control study. Adults with IHCA at a tertiary university hospital were identified, and archived serum samples collected within 48 h before the event were analyzed. For the ROSC analysis, patients with ROSC were matched to non-ROSC patients, while for the IHM analysis, patients with IHM were matched to survivors according to age, sex, initial IHCA rhythm, interval between sample collection and cardiac arrest, and major comorbidities. Untargeted metabolomic profiling was performed, and data was analyzed using complementary multivariate and univariate approaches. Candidate metabolites were further evaluated through qualitative assessment of their distribution across groups. Six metabolites initially differed between groups; however, after this refinement step, only two remained consistently associated with clinically relevant outcomes. Lower circulating tryptophan concentrations were associated with IHM, whereas higher levels of 2-methylbutyryl-L-carnitine were associated with failure to achieve ROSC and increased IHM. Pathway enrichment analysis suggested disruptions in mitochondrial energy metabolism, particularly within the tricarboxylic acid cycle, as well as alterations in amino acid–related pathways, including tryptophan, histidine, and alanine–aspartate–glutamate metabolism. These findings indicate that detectable metabolic disturbances are present up to 48 h before IHCA and are associated with clinical outcomes. As all patients in this study experienced IHCA, these results should be interpreted as differences between outcome groups rather than predictors of IHCA occurrence. Within this context, the findings support the concept that IHCA may represent the culmination of a progressive metabolic deterioration rather than an abrupt event, highlighting a potential window for earlier risk stratification and future investigation of preventive strategies.

**Supplementary Information:**

The online version contains supplementary material available at 10.1038/s41598-026-56981-w.

## Introduction

Cardiac arrest (CA) is defined as the sudden cessation of the heart’s mechanical activity, evidenced by the absence of circulation. CA is typically characterized by location into in-hospital cardiac arrest (IHCA) and out-of-hospital cardiac arrest (OHCA)^1^. Despite prompt medical intervention, survival after IHCA remains dismal, with rates as low as 10%, compared to up to 25% in OHCA^2^. Although IHCA is more accessible for clinical intervention and monitoring, it has been comparatively under-researched, and current treatment guidelines are largely extrapolated from OHCA data^3^.

The investigation of biomarkers represents a promising strategy for elucidating factors that predispose CA and influence prognosis. Biomarkers provide prognostic information and clarify underlying pathophysiological mechanisms. When appropriately integrated into clinical practice, they may improve outcome prediction, support decision-making, and help identify patients most likely to benefit from intensified therapies, thereby optimizing resource allocation and patient care^1^.

Metabolomics enables the simultaneous analysis of numerous metabolites in biological samples. This approach provides insights into biochemical changes associated with disease progression and can identify specific metabolic signatures linked to poor outcomes in CA. However, existing metabolomic studies in CA are limited and have primarily analyzed post-resuscitation samples^4–7^, where ischemia-reperfusion injury (IRI) significantly influences the metabolic profile.

The primary aim of this study was to characterize early plasma metabolic alterations occurring up to 48 h before IHCA. Using routine blood samples obtained before the event, these measurements reflect pre-arrest physiology without the confounding effects of IRI, offering a direct view of metabolic disturbances preceding IHCA. The secondary objective was to evaluate whether these pre-arrest metabolic signatures were associated with return of spontaneous circulation (ROSC) and in-hospital mortality (IHM).

## Methods

### Study design

This exploratory case-control study included patients from an ambispective cohort investigating biomarkers of IHCA^8^ investigated biomarkers of IHCA. The study was conducted at a tertiary university hospital in São Paulo State, Brazil, between August 2021 and April 2023. This study was approved by the Research Ethics Committee of the Botucatu Medical School, São Paulo State University (UNESP), Brazil (protocol number: 46717721.0.0000.5411). All methods were performed in accordance with the relevant guidelines and regulations, including the principles of the Declaration of Helsinki. Written informed consent was obtained from all surviving participants. For patients who died during hospitalization, informed consent could not be obtained due to the nature of the study; in these cases, the requirement for informed consent was waived by the Research Ethics Committee.

### Population

During the study period, all adult patients (≥ 18 years) with IHCA occurring outside intensive care units were screened. In patients with multiple CA events during the same hospitalization, only the first event (defined as the index IHCA) was included in the analysis. Exclusion criteria were: (1) prior OHCA before the index hospitalization; (2) absence of cardiopulmonary resuscitation due to do-not-resuscitate orders; and (3) lack of an archived serum sample collected within 48 h before the event.

Subsequently, eligible patients were divided according to the study outcomes, and two independent matched case-control analyses were performed. For the ROSC analysis, cases consisted of patients who achieved return of spontaneous circulation (ROSC), while controls were patients without ROSC (non-ROSC). For the in-hospital mortality analysis, cases consisted of patients who died during hospitalization (IHM), while controls were patients who survived to hospital discharge (survival). Matched case-control pairs were constructed using a 1:1 strategy for each outcome. Exact matching was performed for sex and initial IHCA rhythm, while age, time interval between sample collection and IHCA, and major comorbidities were matched as closely as possible using a hierarchical approach. Pairs were generated using database tools (Excel) and subsequently reviewed to ensure overall clinical comparability.

### Data collection

Demographic and clinical data were obtained from patient medical records. ROSC was defined as restoring sustained circulation for at least 20 min following CA. Patients were subsequently followed to determine IHM.

### Sample collection and extraction

Archived serum samples from routine clinical draws were processed per institutional protocols, centrifuged shortly after collection, and stored at − 80 °C. Storage time varied, but all samples were kept under identical conditions without prior freeze–thaw cycles and were thawed for metabolomic analysis in August 2023. For extraction, 30 µL of plasma was mixed with 120 µL of cold methanol containing p-fluoro-DL-phenylalanine (2 µg/mL) as internal standard. The mixture was vortexed, sonicated on ice, incubated at − 20 °C for 15 min, and centrifuged at 10,000 ×g for 15 min. The supernatant (120 µL) was evaporated to dryness (Turbovap, 45 °C, 7 psi N₂), reconstituted in 60 µL of methanol: water (3:7), and centrifuged again. A final volume of 50 µL was transferred to vials for analysis. A pooled quality control (QC) sample was prepared and injected periodically to assess system stability.

### Liquid chromatography–high-resolution tandem mass spectrometry analysis and data processing

Liquid chromatography–high-resolution tandem mass spectrometry data were processed in MS-DIAL (RIKEN, v4.9) for signal extraction, deconvolution, and peak alignment using default parameters (MS1: 0.005 Da, MS2: 0.05 Da, peak height ≥ 1.0 × 10⁶, alignment tolerance: 0.3 min/0.005 Da). A blank sample was used to remove background signals. Metabolites were annotated by comparing MS/MS spectra to an in-house library (MassBank of North America + NIST MSMS 2020), retaining matches with similarity > 80% and mass error < 8 ppm (level 2). Detected ions were exported to Excel for data matrix generation. For group comparisons, only features with QC signal variation < 30% were retained.

### Statistical analysis

Demographic and clinical data were analyzed using SigmaPlot for Windows v12.0 (Systat Software Inc., San Jose, CA, USA). Continuous variables are presented as means ± standard deviation (SD) or medians with interquartile ranges (25th–75th percentiles), depending on the distribution. Categorical variables are expressed as frequencies and percentages. Comparisons between the two groups were performed using the Student’s t-test or the Mann–Whitney U test for continuous variables, and the Chi-square test or Fisher’s exact test for categorical variables.

Metabolomic data were analyzed using MetaboAnalyst (v5.0). Considering the limited sample size, both univariate and multivariate approaches were applied. Missing values, assumed to be predominantly below the detection limit (missing not at random, MNAR), were imputed using a low-value replacement strategy, in which missing intensities were replaced by a small fraction of the minimum detected value. This ensured a complete numerical matrix for multivariate analyses. Features with a relative standard deviation > 30% in pooled quality control samples were excluded to ensure analytical robustness. Multivariate analysis included Principal Component Analysis (PCA) and Partial Least Squares Discriminant Analysis (PLS-DA), whereas univariate analysis was performed using Student’s t-test and visualized through volcano plots. To increase confidence in metabolite selection, only features with consistent significance across approaches, high Variable Importance in Projection (VIP) scores, and statistically significant p-values were retained. Multiple testing correction was assessed using False Discovery Rate (FDR) estimation. However, given the exploratory design, biological heterogeneity, and limited sample size, primary significance was defined using raw p-values (*p* < 0.05), and metabolite prioritization was based on an integrative framework combining statistical significance, VIP scores, ROC performance (AUC), and annotation confidence.

Subsequently, violin plots were generated in RStudio Software (v4.4.2) to visualize the distributions of metabolites that differed between groups. This refinement step consisted of a qualitative assessment of metabolite distributions to verify consistent separation between groups, rather than additional statistical filtering. Data preprocessing included median normalization, cubic root transformation, and Pareto scaling to minimize technical variability and improve comparability among samples. Statistical comparisons between outcome groups were conducted using Student’s *t*-test, with *p* < 0.05 considered statistically significant. Finally, a pathway enrichment was performed to identify biologically significant metabolic networks.

## Results

### Cohort characteristics and IHCA events

**Fig. 1:**
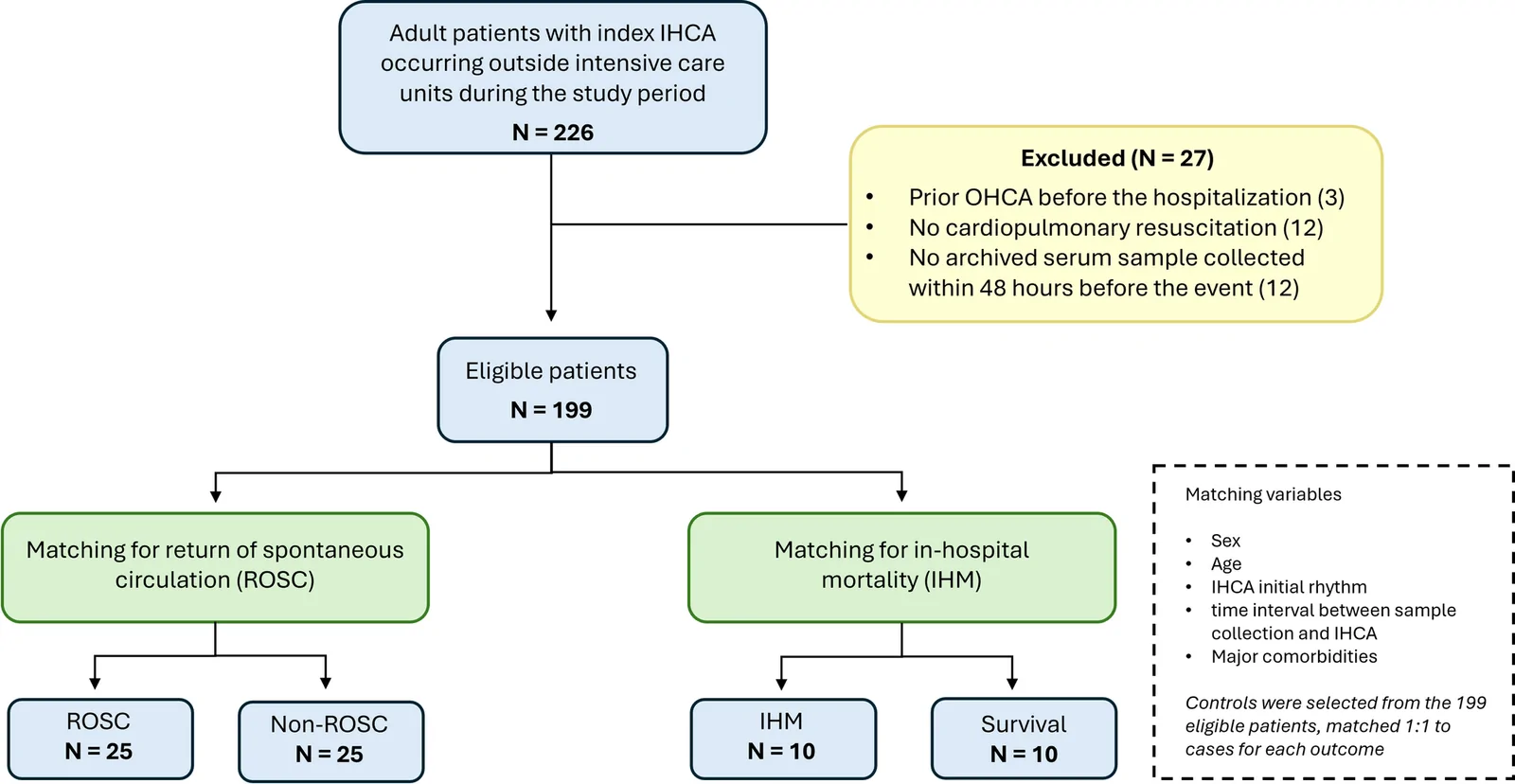
Patient selection and matching flowchart. IHCA = in-hospital cardiac arrest; OHCA = out-of-hospital cardiac arrest.

During the study period, 226 patients with index IHCA occurring outside intensive care units were screened. After applying predefined exclusion criteria, 27 patients were excluded (3 with OHCA, 12 without available serum samples, and 12 who were not resuscitated), resulting in 199 patients included in the final eligible cohort. From this population, matched case-control pairs were constructed for each outcome. For the analysis of ROSC, 25 ROSC patients were matched with 25 non-ROSC patients, and for the analysis of IHM, 10 IHM patients were matched with 10 survivors (Fig. 1). The resulting matched cohorts showed good balance, with no statistically significant differences in baseline characteristics (Table 1).

**Table 1:** Baseline characteristics and laboratory data of patients with in-hospital cardiac arrest.

Variable	ROSC		*p*	In-hospital mortality		*p*
	yes (25)	no (25)		yes (10)	no (10)	
Age (years)	68.0 (59.5–72.0)	68.0 (60.5–73.5)	0.771	61.4 (± 13.2)	60.4 (± 12.0)	0.861
Male gender, n (%)	13 (52)	13 (52)	0.777	9 (90)	9 (90)	1.000
Rhythm, n (%)						
Asystole	10 (40)	10 (40)	1.000	2 (20)	2 (20)	1.000
PEA	10 (40)	10 (40)	1.000	5 (50)	5 (50)	1.000
pVT	2 (8)	2 (8)	1.000	2 (20)	2 (20)	1.000
VF	3 (12)	3 (12)	1.000	1 (10)	1 (10)	1.000
IHCA cause, n (%)						
Cardiac	7 (28)	6 (24)	1.000	2 (20)	5 (50)	0.350
Non cardiac	18 (72)	19 (76)		8 (80)	5 (50)	
IBIS (min)	600 (330–1020)	480 (240–975)	0.690	613,5 (± 319,6)	901,1 (± 873,4)	0.344
Comorbidities, n (%)						
AH	14 (56)	17 (68)	0.435	6 (60)	4 (40)	0.655
DM	10 (40)	13 (52)	0.570	2 (20)	4 (40)	0.628
CKDd	2 (8)	1 (4)	1.000	0 (0)	1 (10)	1.000
Admission, n (%)						
Medical	19 (76)	18 (72)	1.000	9 (90)	8 (80)	1.000
Surgery	6 (24)	7 (28)		1 (10)	2 (20)	
Hemoglobin (g/dL)	11.0 (± 2.9)	11.2 (± 2.5)	0.764	11.6 (± 2.8)	12.7 (± 3.2)	0.463
Urea (mg/dL)	78.0 (37.0–122.3)	89.5 (57.3–147.5)	0.270	104.4 (± 67.5)	58.0 (± 39.0)	0.092
Creatinine (mg/dL)	1.3 (0.8–3.0)	1.7 (0.9–3.7)	0.632	2.2 (0.6–3.6)	0.9 (0.8–2.1)	0.595
Sodium (mmol/L)	139.0 (133.0–141.0)	138.0 (131.0–146.0)	0.587	137.9 (± 7.0)	135.7 (± 6.2)	0.487
Potassium (mmol/L)	4.5 (± 1.0)	4.0 (± 1.0)	0.152	4.2 (± 1.3)	4.4 (± 0.8)	0.762

ROSC = return of spontaneous circulation; PEA: pulseless electrical activity; pVT: pulseless ventricular tachycardia; VF: ventricular fibrillation; AH: arterial hypertension; DM: diabetes; CKDd: chronic kidney disease in dialysis. IBIS: Interval between IHCA and sample collection; min = minutes. Data are expressed as the mean ± standard deviation, median (25–75%), or percentage.

### Pre-arrest metabolic differences before IHCA according to patient outcome

**Fig. 2:**
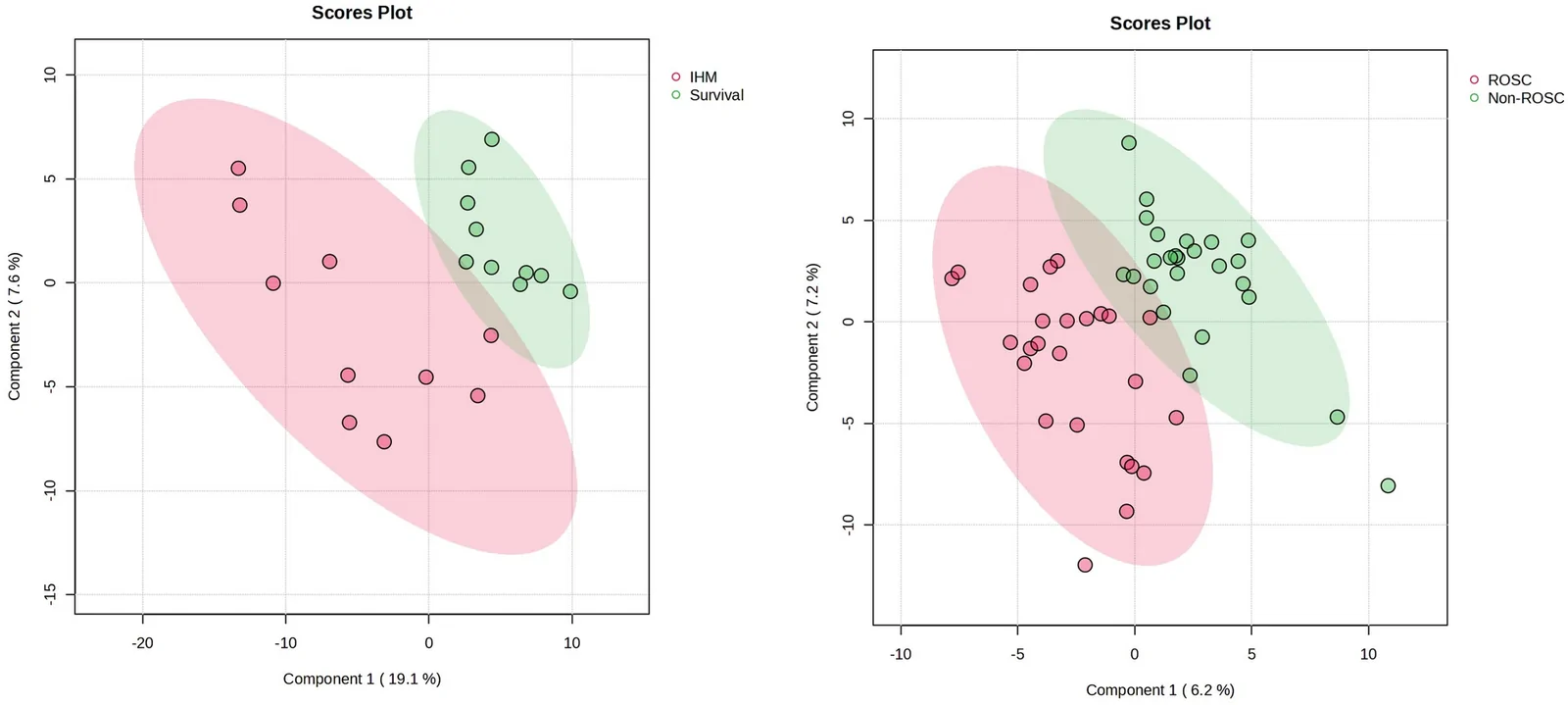
Partial Least Squares Discriminant Analysis (PLS-DA) of metabolomic profiles according to clinical outcomes. [A] IHM vs. Survival groups. [B] Non-ROSC vs. ROSC groups.

Global inspection of metabolomic profiles revealed that patients with IHCA exhibited distinct metabolic patterns based on the likelihood of ROSC and IHM. Supervised PLS-DA analysis distinguished the two study groups (Fig. 2). In parallel, unsupervised PCA provided an overview of global variance across the outcome groups (Supplementary Fig. S1). The complete ranking of metabolites according to Variable Importance in Projection (VIP) scores derived from the PLS-DA models, including direction of change between groups, is provided in the Supplementary Material, ensuring full reporting of multivariate results (Supplementary Table S1, sheets: PLSDA_VIP_IHMxSurvival and PLSDA_VIP_ROSCxN‑ROSC). Corresponding PCA loadings, describing the contribution of each feature to the principal components, are provided in the Supplementary Material (Supplementary Table S1, sheet: PCA_LOADINGS).

**Fig. 3:**
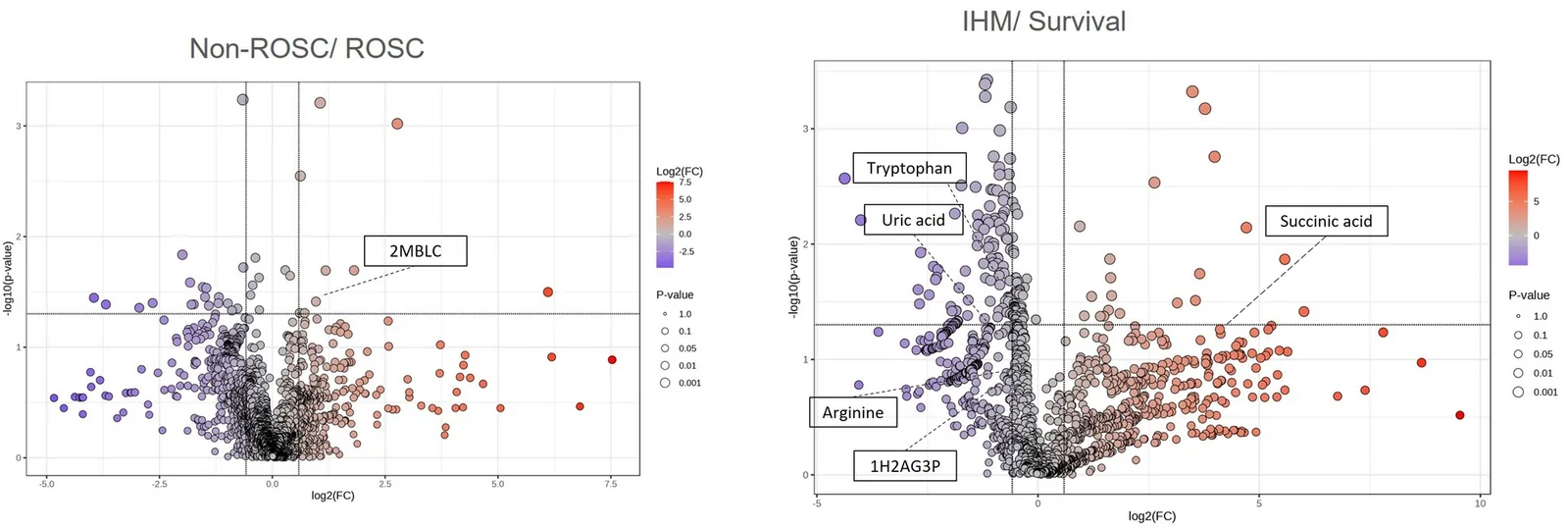
Volcano plot graphs for identification of differentially abundant metabolites. The x-axis represents the magnitude of variation [log2 Fold Change] and the y-axis the statistical significance [-log10 p-value]. [A] Non-ROSC vs. ROSC groups. [B] IHM vs. Survival groups. Red/orange points indicate more abundant metabolites in the Non-ROSC and IHM groups, respectively, while blue/purple points indicate less abundant metabolites. The dashed horizontal line indicates the threshold of statistical significance [*p* = 0.05]. Significantly altered metabolites are directly labeled in the plots to facilitate interpretation.

**Fig. 4:**
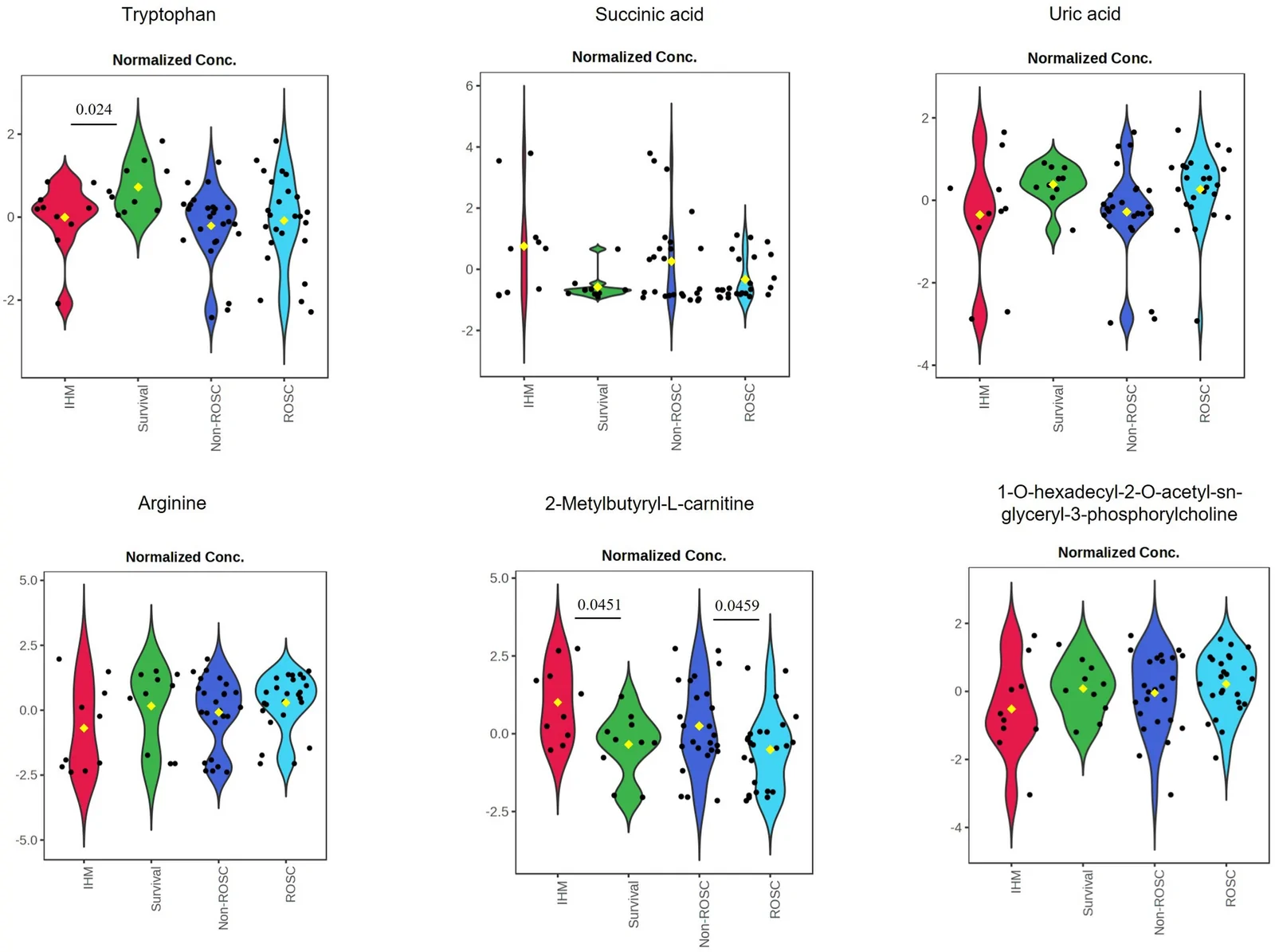
Violin plots of significantly altered metabolites according to clinical outcomes. Comparisons were performed between IHM vs. Survival groups and Non-ROSC vs. ROSC groups.

To further explore potential metabolic differences between outcome groups, we performed a univariate volcano plot analysis to identify individual metabolites exhibiting the largest fold changes combined with statistical significance (Fig. 3). This analysis highlighted six metabolites with potential discriminatory value between groups (Table 2). The corresponding p-values and fold changes for comparisons between survival vs. IHM and ROSC vs. Non-ROSC are summarized in Supplementary Table S2. Complete univariate comparisons between all groups, including feature identifiers, direction of change, log2 fold change, p-values, and ionization mode, are provided in the Supplementary Material (Supplementary Table S1, sheets: Volcano List IHMxSurvivals and Volcano List ROSCxN‑ROSC). These metabolites also demonstrated discriminative performance in receiver operating characteristic analyses (Supplementary Fig. S2). Based on these findings, the selected metabolites were further evaluated using violin plot analysis to assess differences (Fig. 4). After refinement with violin plots, only tryptophan (TRP) and 2-methylbutyryl-L-carnitine (2MBLC) retained statistical significance, whereas other metabolites showed overlapping distributions between groups under standardized preprocessing conditions.

**Table 2:** Identification of discriminating metabolites among plasma samples.

Metabolites	Comparison	Formula	AUC	Concentration level*	Log_2_ FC	*P*
Arginine	IHM* x Survival	C6H14N4O2	0.66	↓	-0.85137	0.13258
Tryptophan	IHM* x Survival	C11H12N2O2	0.76	↓	-0.81706	0.024489
Succinic acid	IHM* x Survival	C4H6O4	0.75	↑	3.2645	0.05907
Uric acid	IHM* x Survival	C5H4N4O3	0.72	↓	-0.67762	0.059296
1H2AG3P	IHM* x Survival	C26H54NO7P	0.65	↓	-0.38318	0.18173
2MBLC	Non-ROSC* x ROSC	C12H23NO4	0.639	↑	0.9728	0.036415

*Concentration level indicated as increased (↑) or decreased (↓) found in the highlighted group* in the “comparison” column. 1H2AG3P = 1-O-Hexadecyl-2-O-acetyl-sn-glyceryl-3-phosphorylcholine; 2MBLC = 2-Methylbutyryl-L-carnitine; FC = Fold Change.

### Associations between pre-arrest metabolites and ROSC

In stratified analyses, 2MBLC was significantly elevated in IHCA patients who failed to achieve ROSC compared with those who achieved ROSC (*p* = 0.0459). No other metabolites demonstrated statistically significant differences after violin plot refinement.

### Associations between pre-arrest metabolites and in-hospital mortality

When IHCA patients were stratified according to in-hospital mortality, TRP concentrations were significantly lower in patients who died compared to those who survived (*p* = 0.024). In contrast, 2MBLC levels were significantly higher in patients with IHM (*p* = 0.0451). Other metabolites did not retain statistical significance after violin plot analysis.

### Pathway enrichment analysis

Pathway enrichment analysis identified disruptions in multiple metabolic pathways before IHCA (Supplementary Fig. S3; Supplementary Table S3). These included the tricarboxylic acid (TCA) cycle as well as amino acid–related pathways such as alanine, aspartate and glutamate metabolism, histidine metabolism, and tryptophan metabolism.

## Discussion

### Summary of major findings

In this study, we characterized metabolic alterations occurring up to 48 h before IHCA. This is the first study to perform metabolomic analysis of pre-arrest blood samples in humans, thereby avoiding the confounding effects of IRI. By combining multivariate, univariate, and pathway-based strategies, we identified a biologically coherent pre-arrest metabolic signature associated with two pre-defined clinical outcomes.

After multistep statistical refinement, two metabolites emerged as outcome-associated markers. Lower TRP concentrations were associated with IHM, whereas elevated 2MBLC was associated with failure to achieve ROSC and IHM. Although other metabolites were highlighted during initial exploratory analyses, they did not retain statistical significance after distribution-based refinement and therefore should be interpreted as preliminary signals rather than definitive predictors of patient outcome.

Pathway analysis further revealed early disruption of the TCA cycle and multiple amino-acid–derived metabolic networks, indicating that mitochondrial energy failure and loss of metabolic flexibility precede circulatory collapse. Together, these findings suggest that IHCA may represent the culmination of a progressive and detectable metabolic decline.

### Comparison with previous studies

Most prior metabolomic studies in CA have focused on post-resuscitation samples, in which IRI, lactate accumulation, and systemic inflammation generate dominant metabolic signals that obscure earlier pathophysiological changes^4–7^. Consequently, these post-ROSC profiles provide limited insight into the metabolic trajectory preceding circulatory collapse.

Our findings extend the current literature by demonstrating that metabolic differences are detectable up to 48 h before IHCA among patients who subsequently experience different clinical outcomes. However, as this study did not include a non-IHCA control group, these findings should be interpreted as differences within IHCA patients, and not predictors of IHCA occurrence. This observation parallels data from sepsis and shock, where progressive metabolic derangement precedes overt clinical deterioration and reflects early mitochondrial stress and impaired metabolic adaptability^9,10^. By characterizing the pre-arrest metabolic landscape in hospitalized patients, this study fills a critical gap in the literature and provides a temporal framework linking silent biochemical deterioration to subsequent CA.

The separation observed between ROSC/non-ROSC and IHM/survival groups further supports the concept that coordinated metabolic signatures, rather than isolated biomarkers, characterize early clinical deterioration. This aligns with prior work showing that metabolomic profiles can outperform single markers in capturing systemic metabolic disorganization during critical illness^11^. In our study, although only TRP and 2MBLC retained statistical significance after distribution-based refinement, the coordinated directional changes observed across metabolites support the presence of a broader, biologically coherent metabolic stress response preceding CA.

### Interpretation of findings

Given the exploratory, case-control design, these results indicate associations rather than causative mechanisms. However, the biological coherence of the pathways identified supports their plausibility. Prospective validation with serial sampling will be necessary to determine causality. Metabolites identified in the exploratory univariate analysis exhibited coordinated directional changes consistent with immunometabolic stress.

Alterations in arginine suggest disrupted nitric oxide bioavailability and potential microcirculatory dysregulation^12^, while increased uric acid reflects enhanced purine turnover and oxidative stress^13^. Changes in succinic acid are compatible with early mitochondrial vulnerability and impaired metabolic handling during hypoxia–reperfusion preceding overt circulatory collapse^14^. In parallel, modulation of ether lipids such as 1-O-Hexadecyl-2-O-acetyl-sn-glyceryl-3-phosphorylcholine points to disturbances in endothelial function or inflammatory signaling^15^. Although these signals did not retain statistical significance after multivariable refinement, their coordinated behavior supports the presence of a broader metabolic response preceding circulatory collapse and warrants further mechanistic investigation. In contrast to these exploratory findings, TRP and 2MBLC remained independently associated with the outcomes after statistical refinement.

TRP is an essential aromatic amino acid obtained exclusively from the diet and serves as a precursor of bioactive metabolites involved in immune regulation, redox balance, and neuroendocrine signaling. Lower circulating TRP levels before IHCA may reflect heightened systemic inflammation and immune dysregulation. In inflammatory states, cytokine-mediated upregulation of indoleamine 2,3-dioxygenase accelerates TRP catabolism along the kynurenine pathway, reducing circulating availability and generating metabolites associated with disease severity and mortality across acute and chronic inflammatory conditions^16^. Beyond its role in the kynurenine pathway, TRP is also a precursor of serotonin and melatonin, which contribute to immune modulation, vascular homeostasis, and cellular stress responses; preservation of higher TRP availability may therefore support greater physiological reserve under critical stress^17^. Consistent with this concept, clinical and experimental studies suggest that adequate TRP availability is associated with improved resilience, reduced mortality, and attenuation of oxidative and inflammatory pathways in vulnerable populations^18,19^. However, given the link between TRP metabolism and systemic inflammation, our findings should be interpreted with caution. As inflammatory markers were not assessed, we could not determine whether the association between TRP and IHM is independent of inflammatory status.

2MBLC is a short-chain acylcarnitine involved in mitochondrial fatty-acid transport and β-oxidation. Accumulation of acylcarnitines reflects impaired mitochondrial substrate utilization and has been linked to oxidative stress, energetic failure, and altered cardiac electrophysiology, particularly in shock and renal dysfunction^20^. Similar acylcarnitine elevations have been described in sepsis, cardiovascular disease, and CA, where they are associated with adverse outcomes^21,22^. Experimental data further indicate that excessive acylcarnitine accumulation exacerbates IRI, whereas limiting their buildup preserves mitochondrial integrity and improves survival^21^. Together, these findings support elevated pre-arrest 2MBLC as a marker associated with mitochondrial dysfunction and adverse outcomes. However, acylcarnitine accumulation has also been linked to chronic metabolic and inflammatory conditions^23^, suggesting that elevated 2MBLC may reflect underlying metabolic vulnerability rather than a process specific to CA.

Pathway enrichment analysis suggested coordinated disruptions across multiple metabolic networks before IHCA, including the TCA cycle and several amino acids–related pathways. However, these findings should be interpreted with caution, as the TCA cycle represents a central and non-specific metabolic hub, and alterations in its intermediates may reflect a common downstream response to diverse metabolic disturbances rather than a pathway uniquely associated with the studied condition^22–27^.

The TCA cycle showed the greatest impact, reflecting early impairment of oxidative phosphorylation. Reduced levels of intermediates such as succinate suggest exhaustion of mitochondrial energetic reserves and diminished capacity to sustain adenosine triphosphate generation under stress^22^. Disruptions in glyoxylate and dicarboxylate metabolism, a compensatory pathway linked to the TCA cycle^23^, likely represent attempts to maintain redox balance during mitochondrial stress; this concomitant enrichment of TCA and glyoxylate/dicarboxylate metabolism has been associated with adverse clinical outcomes in large cardiovascular cohorts^24^. These findings point to an early loss of metabolic flexibility, consistent with evidence that mitochondrial dysfunction is an initial and potentially reversible phase in the progression toward multi-organ failure.

Alterations in TRP and histidine metabolism suggest activation of amino-acid-derived redox and immune pathways, with increased kynurenine-pathway flux generating reactive and immunomodulatory metabolites that may exacerbate endothelial and neuronal injury^26^, while disturbed histidine catabolism may reflect an insufficient antioxidant response^27^. Finally, disruptions in alanine, aspartate, and glutamate metabolism indicate increased transamination activity and nitrogen imbalance, metabolic features commonly seen in catabolic and hypoxic states preceding cardiovascular collapse^28^.

### Implications for clinicians and researchers

The identification of a measurable metabolic signature up to 48 h before IHCA highlights a potentially clinically actionable window that is currently underrecognized. These findings suggest that combining targeted metabolite panels with conventional early warning systems may enhance risk stratification by capturing biochemical deterioration before overt clinical instability. While untargeted metabolomics remains impractical for bedside use, targeted LC-MS/MS assays capable of quantifying key metabolites such as succinic acid and acylcarnitines could be implemented more feasibly in critical-care settings^29^.

Therapeutically, these findings raise the possibility that early interventions aimed at restoring mitochondrial oxidative metabolism, reducing acylcarnitine accumulation or modulating kynurenine-pathway activation could attenuate metabolic decline and potentially alter the clinical trajectory before CA occurs. By reframing IHCA as the end stage of a detectable and potentially modifiable metabolic deterioration, this study opens avenues for preventive metabolic monitoring and early intervention strategies.

### Study strengths and limitations

This study has limitations inherent to its exploratory, case-control design, including sample dispersion and a modest sample size, which limit generalizability, preclude causal inference, and underscore the need for an independent validation phase to confirm the robustness of the findings and to better elucidate differences in metabolite abundance between groups. Other limitations include the unavailability of data on medication use and information on whether CA events were witnessed, raising the possibility of residual confounding. Subgroup analyses according to IHCA rhythm were not feasible due to the limited sample size. Although rhythm was included as a matching variable, residual differences in underlying pathophysiology may still influence metabolomic profiles. Despite these limitations, the study presents important strengths, including the innovative use of pre-cardiac arrest samples, the rigorous matching of essential clinical variables, and the application of complementary multivariate, univariate, and metabolic pathway-based analytical approaches. By focusing on samples collected before circulatory collapse, this study offers a unique perspective on the biological processes preceding IHCA.

### Areas for future research

Future research should focus on comparing metabolomic signatures between patients experiencing IHCA and control patients without IHCA. Future studies should also account for additional confounding variables such as medication use, inflammatory markers, and the presumed etiology of IHCA, particularly the occurrence of acute coronary syndromes.

## Conclusion

This study shows that distinct metabolic disturbances are detectable up to 48 h before IHCA. Our findings support the concept that IHCA represents the culmination of progressive metabolic deterioration rather than an abrupt event. Although exploratory, these results highlight a clinically relevant pre-arrest window that may enable improved risk stratification of IHCA outcomes and the development of preventive metabolic strategies.

## Supplementary Information

Below is the link to the electronic supplementary material.

Supplementary Material 1

Supplementary Material 2

Supplementary Material 3

## Data Availability

Due to institutional policy, the datasets generated and/or analyzed during the current study are not publicly available but are available from the corresponding author upon reasonable request.
