# Supplementary material for: Metabolomic signatures prior to in-hospital cardiac arrest: insights into potential therapeutic targets
Source: Sci Rep. 2026 Jun 12;16:26865. doi: 10.1038/s41598-026-56981-w (PMC13518925; doi:10.1038/s41598-026-56981-w)
Supplement: Supplementary file 1 [file 41598_2026_56981_MOESM1_ESM.docx]

**Supplementary Figure S1.** PCA score plot. Visualization of the variability in the acquired data and assessment of analytical stability through the evaluation of QC samples throughout the analyses.

Arginine

Tryptophan

- Uric acid

1-O-Hexadecyl-2-O-acetyl-sn-glyceryl-3-phosphorylcholine

- Succinic acid

2-Methylbutyryl-L-carnitine

Uric acid

Figure S2- ROC curves of the six annotated metabolites and their respective boxplots.

**Supplementary Figure S2.** ROC curves of the six annotated metabolites and their respective boxplots.

**Supplementary Figure S3.** Pathway Analysis visualization. The graph demonstrates the impact [x-axis] and statistical significance [y-axis, -log10 p-value] of the altered metabolic pathways. The circle size and color indicate the pathway's impact and significance, respectively.
