# Supplementary material for: Metabolomic signatures prior to in-hospital cardiac arrest: insights into potential therapeutic targets
Source: Sci Rep. 2026 Jun 12;16:26865. doi: 10.1038/s41598-026-56981-w (PMC13518925; doi:10.1038/s41598-026-56981-w)
Supplement: Supplementary file 2 [file 41598_2026_56981_MOESM3_ESM.docx]

**Supplementary Table S2.** Differential metabolite abundance between IHM vs. Survival and Non-ROSC vs. ROSC groups.

| Metabolites | p-value survival x IHM | p-value NonROSC x ROSC | Log2FC Survival x IHM | Log2FC NonROSC x ROSC |
| --- | --- | --- | --- | --- |
| Tryptophan | 0.024489 | 0.6532 | -0.81706 | -0.2362 |
| 2MBLC | 0.045161 | 0.0459 | 0.98457 | 0.9728 |
| Succinic acid | 0.05907 | 0.0801 | 3.2645 | 2.0667 |
| Uric acid | 0.059296 | 0.0639 | -0.67762 | -0.4195 |
| Arginine | 0.13258 | 0.2874 | -0.85137 | -0.2103 |
| 1H2AG3P | 0.18173 | 0.3619 | -0.38318 | -0.1127 |

Legends: 1H2AG3P: 1-O-Hexadecyl-2-O-acetyl-sn-glyceryl-3-phosphorylcholine; 2MBLC: 2-Methylbutyryl-L-carnitine

**Supplementary Table S3.** Pathway analysis match results.

| Pathway Name | Match Status |
| --- | --- |
| [Citrate cycle](https://www.metaboanalyst.ca/Secure/pathway/PathResultView.xhtml) (TCA [cycle)](https://www.metaboanalyst.ca/Secure/pathway/PathResultView.xhtml) | [2/20](https://www.metaboanalyst.ca/Secure/pathway/PathResultView.xhtml) |
| Alanine[,](https://www.metaboanalyst.ca/Secure/pathway/PathResultView.xhtml) aspartate and glutamate metabolism | [2/28](https://www.metaboanalyst.ca/Secure/pathway/PathResultView.xhtml) |
| Arginine [biosynthesis](https://www.metaboanalyst.ca/Secure/pathway/PathResultView.xhtml) | [1/14](https://www.metaboanalyst.ca/Secure/pathway/PathResultView.xhtml) |
| Butanoate [metabolism](https://www.metaboanalyst.ca/Secure/pathway/PathResultView.xhtml) | [1/15](https://www.metaboanalyst.ca/Secure/pathway/PathResultView.xhtml) |
| Histidine metabolism | 1[/16](https://www.metaboanalyst.ca/Secure/pathway/PathResultView.xhtml) |
